# Supplementary material for: Causal Relationship between Aging and Anorexia Nervosa: A White-Matter-Microstructure-Mediated Mendelian Randomization Analysis
Source: Biomedicines. 2024 Aug 16;12(8):1874. doi: 10.3390/biomedicines12081874 (PMC11351342; doi:10.3390/biomedicines12081874)
Supplement: Supplementary file 1 [file biomedicines-12-01874-s001.zip › Supplementary Figure/Figure S4/Singe SNP plot of MR analysis for TL to ubm-b-1470.pdf]

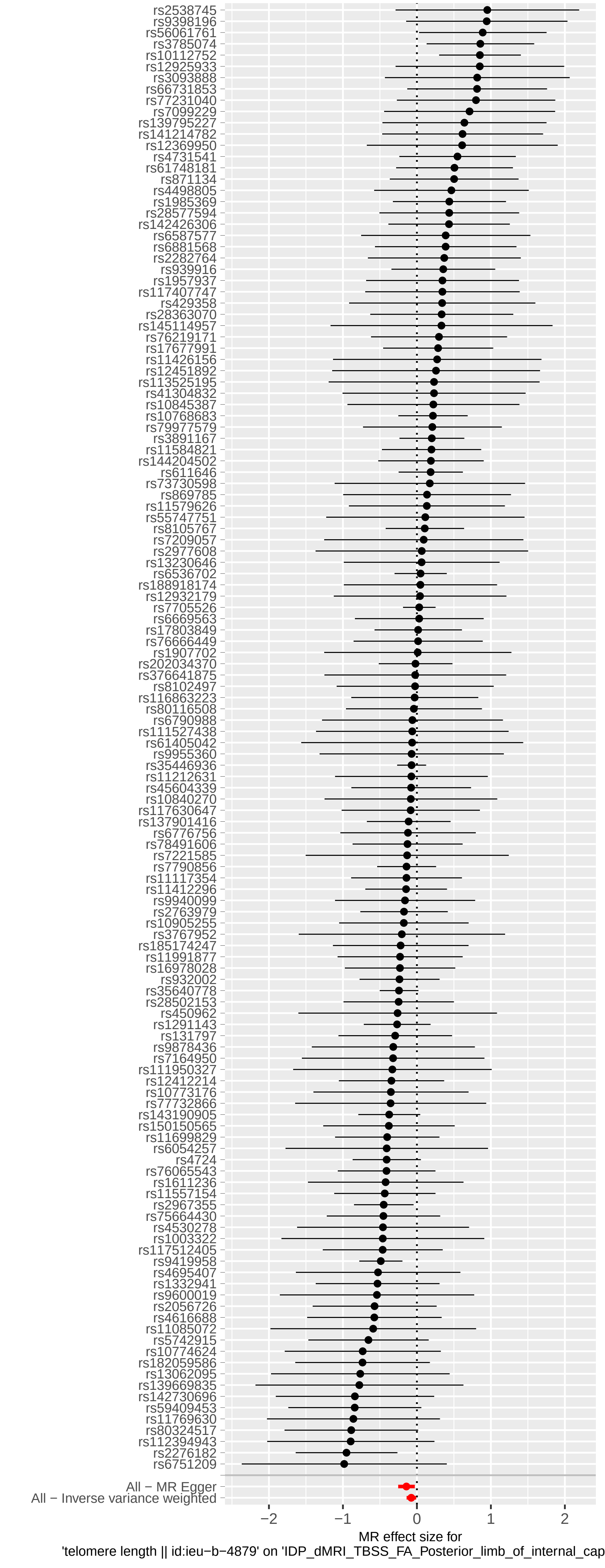
rs2538745
rs9398196
rs56061761
rs3785074
rs10112752
rs12925933
rs3093888
rs66731853
rs77231040
rs7099229
rs139795227
rs141214782
rs12369950
rs4731541
rs61748181
rs871134
rs4498805
rs1985369
rs28577594
rs142426306
rs6587577
rs6881568
rs2282764
rs939916
rs1957937
rs117407747
rs429358
rs28363070
rs145114957
rs76219171
rs17677991
rs11426156
rs12451892
rs113525195
rs41304832
rs10845387
rs10768683
rs79977579
rs3891167
rs11584821
rs144204502
rs611646
rs73730598
rs869785
rs11579626
rs55747751
rs8105767
rs7209057
rs2977608
rs13230646
rs6536702
rs188918174
rs12932179
rs7705526
rs6669563
rs17803849
rs76666449
rs1907702
rs202034370
rs376641875
rs8102497
rs116863223
rs80116508
rs6790988
rs111527438
rs61405042
rs9955360
rs35446936
rs11212631
rs45604339
rs10840270
rs117630647
rs137901416
rs6776756
rs78491606
rs7221585
rs7790856
rs11117354
rs11412296
rs9940099
rs2763979
rs10905255
rs3767952
rs185174247
rs11991877
rs16978028
rs932002
rs35640778
rs28502153
rs450962
rs1291143
rs131797
rs9878436
rs7164950
rs111950327
rs12412214
rs10773176
rs77732866
rs143190905
rs150150565
rs11699829
rs6054257
rs4724
rs76065543
rs1611236
rs11557154
rs2967355
rs75664430
rs4530278
rs1003322
rs117512405
rs9419958
rs4695407
rs1332941
rs9600019
rs2056726
rs4616688
rs11085072
rs5742915
rs10774624
rs182059586
rs13062095
rs139669835
rs142730696
rs59409453
rs11769630
rs80324517
rs112394943
rs2276182
rs6751209
All − MR Egger
All − Inverse variance weighted
−2
−1
0
1
2
MR effect size for
'telomere length || id:ieu−b−4879' on 'IDP_dMRI_TBSS_FA_Posterior_limb_of_internal_cap
